# Modeling Search Behaviors during the Acquisition of Expertise in a Sequential Decision-Making Task

**DOI:** 10.3389/fncom.2017.00080

**Published:** 2017-09-08

**Authors:** Cristóbal Moënne-Loccoz, Rodrigo C. Vergara, Vladimir López, Domingo Mery, Diego Cosmelli

**Affiliations:** ^1^Department of Computer Science, School of Engineering, Pontificia Universidad Católica de Chile Santiago, Chile; ^2^Facultad de Medicina, Biomedical Neuroscience Institute, Universidad de Chile Santiago, Chile; ^3^Center for Interdisciplinary Neuroscience, Pontificia Universidad Católica de Chile Santiago, Chile; ^4^School of Psychology, Pontificia Universidad Católica de Chile Santiago, Chile

**Keywords:** sequential decision-making, Hidden Markov Models, expertise acquisition, behavioral modeling, search strategies

## Abstract

Our daily interaction with the world is plagued of situations in which we develop expertise through self-motivated repetition of the same task. In many of these interactions, and especially when dealing with computer and machine interfaces, we must deal with sequences of decisions and actions. For instance, when drawing cash from an ATM machine, choices are presented in a step-by-step fashion and a specific sequence of choices must be performed in order to produce the expected outcome. But, as we become experts in the use of such interfaces, is it possible to identify specific search and learning strategies? And if so, can we use this information to predict future actions? In addition to better understanding the cognitive processes underlying sequential decision making, this could allow building adaptive interfaces that can facilitate interaction at different moments of the learning curve. Here we tackle the question of modeling sequential decision-making behavior in a simple human-computer interface that instantiates a 4-level binary decision tree (BDT) task. We record behavioral data from voluntary participants while they attempt to solve the task. Using a Hidden Markov Model-based approach that capitalizes on the hierarchical structure of behavior, we then model their performance during the interaction. Our results show that partitioning the problem space into a small set of hierarchically related stereotyped strategies can potentially capture a host of individual decision making policies. This allows us to follow how participants learn and develop expertise in the use of the interface. Moreover, using a Mixture of Experts based on these stereotyped strategies, the model is able to predict the behavior of participants that master the task.

## 1. Introduction

Whether you are preparing breakfast or choosing a web link to click on, decision making processes in daily life usually involve sequences of actions that are highly dependent on prior experience. Consider what happens when you interact with an ATM machine: you have to go through a series of specific button presses (i.e., actions) that depend on whether you are interested in, for instance, drawing money or consulting your account balance (i.e., the outcome). Despite some commonalities, which sequence you use will depend on the specific ATM brand you are dealing with, while previous exposure will determine which behavioral strategy you deploy. Maybe you cautiously explore the available choices and hesitate before pressing each button; maybe this is the same machine you have used for the last year, so you deftly execute a well practiced sequence of actions to draw some cash.

Sequential choice situations such as the ATM example are pervasive in everyday behavior. Not surprisingly, its importance for the understanding of human decision making in real-world scenarios has been recognized for long time, as a wealth of studies attest (Rabinovich et al., 2006; Gershman et al., 2014; Otto et al., 2014; Cushman and Morris, 2015; see Walsh and Anderson, 2014 for review). Yet, despite its importance, the issue of how expertise is developed in the context of sequential choice situations remains still under-explored. While the study of optimal strategies or courses of action to solve sequential choice scenarios is a fundamental aim of such studies (Alagoz et al., 2009; Friedel et al., 2014; Schulte et al., 2014; Sepahvand et al., 2014), it is still necessary to better understand how agents learn and acquire such strategies as they interact with the world (Fu and Anderson, 2006; Acuña and Schrater, 2010; Sims et al., 2013).

Human-computer interfaces offer a privileged scenario to study the development of expertise in sequential decision making processes. As we learn to use an interface, isolated exploratory actions turn into expert goal-directed sequences of actions by repeatedly testing and learning to adjust behavior according to the outcome (Solway and Botvinick, 2012). Furthermore, such scenarios are particularly well adapted to sampling behavioral data in varying degrees of ecological validity. They also allow for testing different ways to use such behavioral data to make the interface more responsive. Indeed, in addition to contributing to a better understanding of the psychological and neurobiological mechanisms underlying sequential decision making, taking into account how people learn to interact with novel systems could have relevant consequences for adaptive interface design. Here we tackle the question of expertise acquisition in a sequential decision making scenario. We aim to discover if individuals can be described in terms of specific behavioral strategies while they learn to solve the task and, if so, whether this can be used to predict future choices.

Several approaches have been developed to address the problem of modeling behavior in sequential decision making scenarios. The Reinforcement Learning (RL) paradigm has been successfully extended to model behavior during sequential choice tasks (Dayan and Niv, 2008; Acuña and Schrater, 2010; Dezfouli and Balleine, 2013; Daw, 2014; Walsh and Anderson, 2014). In general terms, RL techniques aim at finding a set of rules that represent an agent's policy of action given a current state and a future goal by maximizing cumulative reward. Because actions are chosen in order to maximize reward, it is necessary to assign value to the agent's actions. Reward schemes work well when gains or losses can be estimated (e.g., monetary reward). However, in many of our everyday interactions, reward in such an absolute sense is difficult to quantify. Accordingly, the accuracy of an arbitrary reward function could range from perfect guidance to totally misleading (Laud, 2004).

To overcome the difficulty of defining a reward function, the Inverse Reinforcement Learning based approaches try to recover a reward function from execution traces of an agent (Abbeel and Ng, 2004). Interestingly, this technique has been used to infer human goals (Baker et al., 2009), developing into methods based on Theory of Mind to infer peoples' behavior through Partially Observable Markov Decision Process (POMDP) (Baker et al., 2011). Although, these techniques are important steps in the area of plan recognition, they usually focus on which is the best action an agent can take given a current state, rather than determine high-level patterns of behavior. Also, they consider rational agents who think optimally. However, in learning scenarios, optimal thinking is achieved through trial and error, rather than being the de facto policy of action.

Alternative modeling approaches exist that do not require defining a reward function to determine behaviors. Specifically, Markov models have been adapted to analyze patterns of behavior in computer interfaces, such as in web page navigational processes (Ghezzi et al., 2014; Singer et al., 2014), were no simple, unitary reward is identifiable. In general terms, Markov models are aimed at modeling the stochastic dynamics of a system which undergoes transitions from one state to another, assuming that the future state of the system depends only on the current state (Markov property). For example, a Markov chain of navigational process can be modeled by states representing content pages and state transitions representing the probability of going from one page to another (e.g., going from the login page to the mail contacts or to the inbox). Possible behaviors or cases of use can then be extrapolated from the structure of the model (see Singer et al., 2014 for an example). In general, however, these behaviors are highly simplified descriptions of the decision-making process because they do not consider the rationale behind the user's actions, and only focus on whether the behavioral pattern is frequent or not. Accordingly, if the user scrolls down the page searching for a specific item in some order and then makes a decision, the psychological processes behind his or her actions are ignored.

An interesting extension of the simpler Markov models, which aims to capture the processes underlying decision making behavior, are the Hidden Markov Models (HMM) (Rabiner, 1989). In a HMM, the states are only partially observable and the nature of the underlying process is inferred only through its outcomes. This relationship between states and outcomes allows modeling a diversity of problems, including the characterization of psychological and behavioral data (Visser et al., 2002; Duffin et al., 2014). Of special interest is the use of HMMs to model the strategic use of a computer game interface (Mariano et al., 2015). Using sets of HMMs, Mariano and collaborators analyze software activity logs in order to extrapolate different heuristics used by subjects while they discover the game's rules. Such heuristics, which are extrapolated a posteriori, are represented by hidden states composed by the grouping of actions and the time taken to trigger such actions. These are then used to identify patterns of exploratory behavior and behaviors representative of the mastery of the game. Interestingly, such heuristics show a good adjustment with self-reported strategies used by the participants throughout the task (Mariano et al., 2015).

Generally speaking, however, Markov models use individual actions to represent hidden states (such as click this or that icon) and more complex high-level behavioral heuristics (such as policies of action) are only inferred after the experimental situation or setup (see Mariano et al., 2015). This represents a potential limitation if we are to build interfaces that are responsive to the learning process as it unfolds. Indeed, throughout the acquisition of different skills, there is abundant evidence that humans and other animals rely on grouping individual actions into more complex behavioral strategies such as action programs or modules to achieve a certain goal (Marken, 1986; Manoel et al., 2002; Matsuzaka et al., 2007; Rosenbaum et al., 2007). This can happen while individual actions remain essentially unchanged and only the way they are organized changes. For instance, it has been shown that throughout the development of sensorimotor coordination, individual movements progressively become grouped into sequences of movements, as the child becomes adept at controlling goal-directed actions (Bruner, 1973; Fischer, 1980).

If expertise development and skill acquisition implies the hierarchical organization of individual actions into complex, high-level behavioral strategies, taking this into account could represent a relevant line of development for behavioral modeling. Recently, this hierarchical structure of behavior has been considered in some approaches, such as Hierarchical Reinforcement Learning (HRL) (Botvinick, 2012; Botvinick and Weinstein, 2014). The idea behind HRLs is to expand the set of actions available to an agent to include a set of extended high-level subroutines which coordinate multiple low-level simple actions that otherwise the agent would have to execute individually. An interesting consequence of this is that it could potentially aid in reducing the dimensionality of the problem, a critical issue in behavioral modeling (Doya and Samejima, 2002; Botvinick, 2012; Shteingart and Loewenstein, 2014). Furthermore, if flexibility in membership between low-level actions and behaviors exist, modeling behaviors in a probabilistic way could help in better capturing the dynamical nature of expertise acquisition.

In this work we are interested in modeling the behavior of users confronted with a sequential decision-making task with limited feedback, in a way that sheds light into potentially relevant learning strategies. While our short term goal is to be able to consistently reproduce search behaviors that individual users exhibit, our long term goal is to inform the construction of interfaces that can adapt to different learning curves. In the following Method Section, we will first present the overall rationale for the approach. We will then describe the experimental task used to test the model and the characteristics of the participants. The bulk of the section is then dedicated to presenting the modeling framework in detail. Results are then presented mainly in terms of the performance of the model for different types of participants as well as in terms of its capacity to predict their behavior. We end with a discussion of our approach as well as its limitations and potential further developments.

## 2. Methods

### 2.1. Rationale of the approach

We present here a HMM-based approach that capitalizes on the hierarchical structure of behavior to model the performance of individuals as they develop expertise in a sequential decision making task that is structured as a 4-level Binary Decision Tree (BDT). We use the HMM structure to infer the distribution of probabilities of a modular set of pre-defined stereotyped high-level strategies (represented by hidden states), while observing the outcome of the user's actions. As such, this approach is reminiscent of type-based methods where one focuses on a pre-specified group of behaviors among all possible behaviors (Albrecht et al., 2016). Such “types” of behavior can be hypothesized based on previous knowledge of the interaction or on the structure of the problem. In our case, pre-defined strategies (i.e., decision-making policies) are selected in order to cover a series of increasingly efficient behaviors in the context of the BDT: from random unstructured exploration to goal-directed actions driven by feedback and knowledge of the task's structure. Additionally, this approach allows us to use the modular architecture as a Mixture of Experts (see Doya and Samejima, 2002 for a similar idea). This enables us to model the evolution of the user's behavior while simultaneously asking the experts about the user's most probable next choice. As a consequence, it is possible to evaluate the model also in terms of it's capacity to predict future actions, which would be desirable in the context of adaptive interface design.

**Figure 1:**
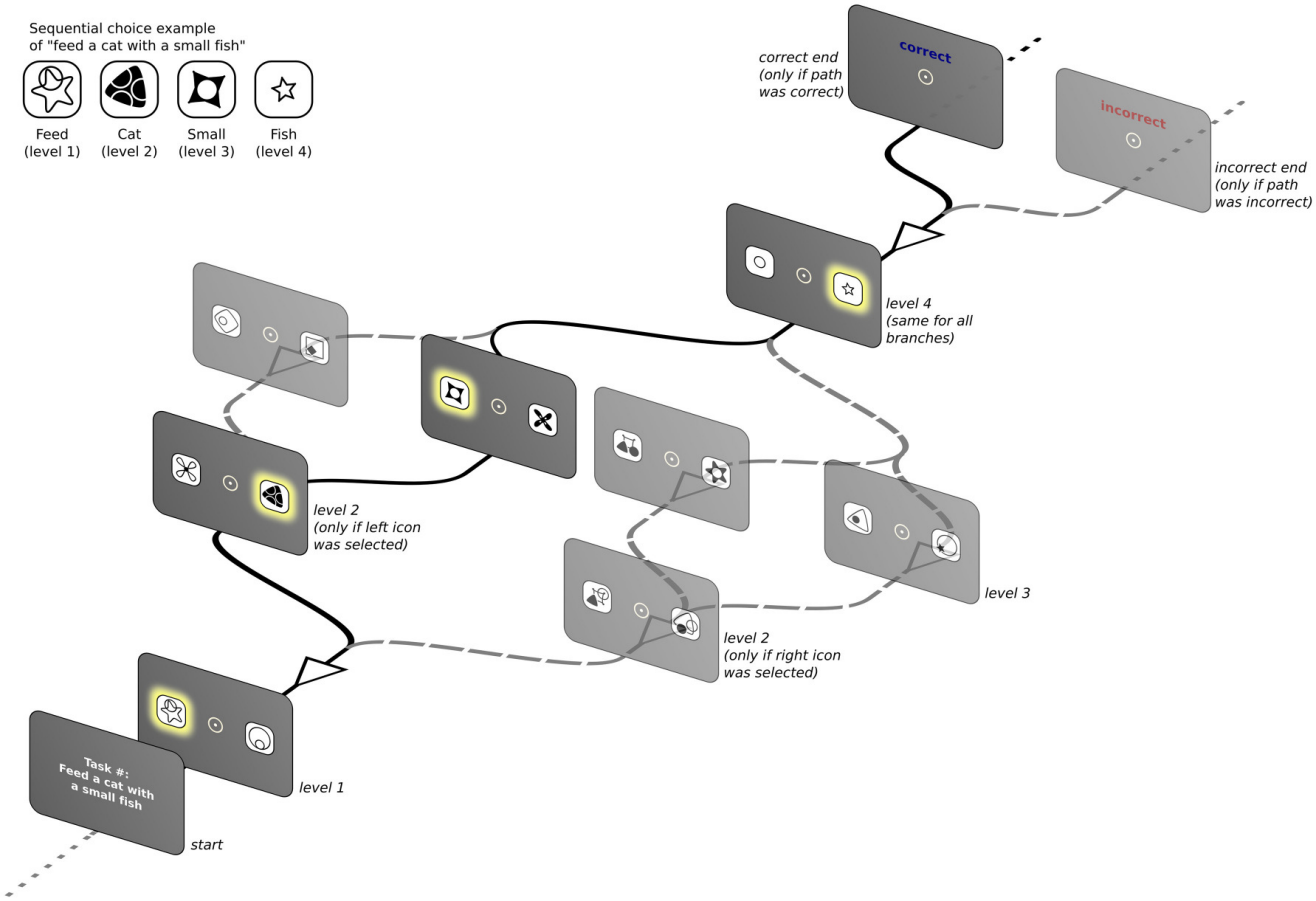
Schematic presentation of the BDT showing one possible instance of the task. Only one screen per level is presented, depending on the icon clicked previously. Highlighted icons along the black continuous lines represent the correct icon-to-concept mapping (see inset) that, when clicked in the correct sequence, produces a positive feedback.

We test this approach using a simple computational interface game where an underlying concept-icon mapping must be discovered in order to complete the task. As mentioned above, the game is structured as a four-level BDT with limited feedback (see Figure 1; see also Fu and Anderson, 2006, p. 198 for a structurally similar design). Each node of the tree represents a decision, and the links between nodes represent the consequences of each decision. The depth of the tree represents the number of sequential choices that are needed to achieve a specific goal. This scenario captures sequential decision-making situations with limited feedback that are pervasive in real world human-computer interface interaction. Furthermore, it allows us to follow the development of expertise as the users discover the rules of the game and deploy different search strategies to solve the task.

### 2.2. Task structure

Figure 1 presents a schematic depiction of the task interface structure that instantiates the BDT. Participants were presented with a computer screen that had an instruction of the type “*verb*_L1_ with a *noun*_L2_ an *adjective*_L3_ *noun*_L4_” (i.e., “*Feed a cat with a small fish*,” see inset in Figure 1). Note that because the task was performed by native Spanish speakers, the the final adjective-noun pair is inverted regarding the previous instruction example and would read “*Alimenta un gato con un pez pequeño.”* After clicking anywhere on this first screen, participants were confronted with the first binary choice (*level 1* of the BDT) with two icons located 2.4° to each side of a central fixation spot. Each of the variable words in the instruction had a fixed mapping to an abstract icon and level of the BDT (as indexed by subscripts in the instruction example above), but participants were not informed of this fact. They were instructed to click using the computer mouse on one of the two icons to proceed to the next screen where the next set of two icons was presented (*level 2* of the BDT). The overall arrangement of the icons remained constant and only their identity changed according to the specific task mapping. This branching structure was repeated until level 4 was reached. The last level was always the same regardless of the branch because only two possible adjectives were used: “small (pequeño)” or “large (grande).” Once subjects clicked an icon in level 4 they received feedback informing them whether the path they had chosen was correct. In the case of a negative feedback, subjects had no way of knowing, a priori, at which level they had made the wrong choice. Therefore, they had to discover the mapping based exclusively on their exposure to successive iterations of the task and the feedback received at the end of each chosen path. Each instruction (corresponding to a single task instance) was presented repeatedly until the participant was able to choose the correct path 5 times in a row before moving onto the next possible instruction. After 15 successive wrong answers, participants were asked whether they wanted to move onto the next task. If they refused, after 10 further wrong answers they were asked again. If they decided to persist in the same task, they only had 5 additional chances to find the correct path else they were forced to move onto the next one. The maximum time allowed to complete the entire task was set to 40 min.

### 2.3. Participants

Twenty-two participants were recruited (12 females) of ages ranging from 20 to 32 years old, with a mean age of 26 ± 3 years (mean ± *SD*). All participants reported normal or corrected-to-normal vision and no background of neurologic or psychiatric conditions. Participants performed on average 162 ± 51 trials (mean ± *SD*) during the course of the experiment (range 89–325). Trials are defined as repetitions of an instance of the task, from the instruction to the feedback after the sequence of choices. Each task instance contains on average 10.79 ± 1.47 trials (mean ± *SD*).

The study was approved by the Ethics Committee of the School of Psychology of the Faculty of Social Sciences, Pontificia Universidad Católica de Chile. All participants gave written informed consent. Twenty-two subjects participated in this study and the nature of the task was explained to all upon arrival to the Laboratory. All experiments were performed in the Psychophysiology Lab of the School of Psychology of the same University. Participants sat in a dimly illuminated room, 60 cm away from a 19-inch computer screen with a standard computer mouse in their right hand. All participants were right handed. Prior to staring the task, subjects were fitted with a 32-electrode Biosemi ActiveTwo © digital electroencephalographic (EEG) system, including 4 electrooculographic (EOG) electrodes, two of them placed in the outer canthi of each eye and two above and below the right eye. Continuous EEG was acquired at 2,048 Hz and saved for posterior analysis. Throughout the task, participants were instructed to maintain fixation on a central spot in order to avoid eye-movement related artifacts in the EEG data. We do not report here the analysis of electrophysiological recordings and will limit ourselves to the behavioral data. All stimuli were presented around fixation or 2.4° to each side of the central fixation spot. This ensured that, despite the instruction to maintain fixation, all participants could easily see all stimuli and perform the task without difficulty in perceptual terms.

### 2.4. Modeling framework

#### 2.4.1. Low-level actions vs. strategies

The simplest action that can be taken on the BDT is to click one of the two possible icons of the binary choice. We will therefore consider these two as the only low level actions for the model. What such low level actions mean or represent, in terms of learning of the BDT, depends on whether the participant is using them in some systematic way to obtain positive feedback (i.e., a strategy). We consider such systematic combination of low-level actions the high-level strategies of the model.

Different strategies can be used to explore and learn the structure of the BDT. In the following we model four high-level decision-making policies in the form of well- defined search strategies, that account for increasingly sophisticated ways to solve the task:

1. **Random Search Strategy:** If the participant displays no systematic use of low level actions, we label this as random behavior. In other words, overt actions seem to be unrelated to the task's demands so that we can only assume ignorance regarding the underlying decision making strategy.
2. **Spatial Search Strategy:** If the participant shows evidence of acting based exclusively on information regarding the spatial layout of the BDT, regardless of the identity of the presented icons (for instance, by choosing to explore from the leftmost to the rightmost branch), we label this as a spatial behavior. When clicking based on spatial features, the participant iteratively discards paths of the BDT so that complete knowledge can be obtained only when the 16 paths of the BDT are correctly recognized. Accordingly, as paths share common information, the learning curve of a user invested exclusively in this strategy will grow exponentially as the search space becomes smaller.
3. **Generative Search Strategy:** If the participant shows evidence of considering the identity of individual icons to guide her choice of actions to reach positive feedback, we label this as a generative behavior. In other words, it implies a first level of successful mapping between current task instruction and the specific BDT instance that is being explored (i.e., when the participant learns that a given icon means a given concept). When clicking based on generative relationships, the participant discards subtrees of the BDT where it is not possible to reach positive feedback. Complete knowledge of the BDT can be obtained when the 16 icons are correctly mapped. All the generative relationships can be learned by being exposed to positive feedback in the 8 paths that contains all of them. Therefore, the learning curve of this strategy is represented by a sigmoid function.
4. **Discriminative Search Strategy:** Here the participant uses a generative model, but adds the ability to learn and relate the negative form of a concept-icon relationship (i.e., learn A by a generative association and then label the neighbor as not-A). In other words, the discriminative search strategy is one that predicts concepts that have not yet been seen in the scope of positive feedback. Concepts are deduced from the context and the understanding of the rules of how the interface works. When clicking based on discriminative relationships, the participant can prune the BDT subtrees more aggressively to obtain positive feedback. As in the generative case, complete knowledge of the BDT can be obtained when the 16 icons are correctly mapped. However, all discriminative relationships can now be learned by being exposed to positive feedback in the 4 paths that contains all of them. Accordingly, the learning curve of this strategy is represented by a sigmoid function that is steeper than in the generative case.

Each of the above models has an initial domain of action that corresponds to the set of actions that can be performed on the BDT according to the strategy's rules. Once exploration of the interface is underway, the initial domain of action of each strategy will necessarily change. This can happen because the user learns something about the specific task instance he is currently solving, or because he learns something about the overall structure of the interface. It is therefore necessary to define criteria that, according to each strategy's rules, allow one to update their domain of action depending on local (task-instance) and global (task-structure) knowledge. Local updates criteria will coincide with the rules of the spatial strategy for all systematic strategies, because according to our hierarchical definition, the simplest way to discard places of the BDT systematically is using spatial information. Conversely, for global updates—and for the sake of simplicity—we will define knowledge in terms of optimal behavior, (i.e., learning places or concepts of the task instances by repeating the correct path 5 times in a row). Table 1 presents the formal definition of the domain of action *D*_*i*_ and both updating schemes for each strategy *s*_*i*_.

**Table 1:** Formal definitions of the strategies are presented according to their initial domain, domain of action updates (task instance learning), and knowledge updates (task structure learning).

Initialization of the domain of action (defines *D*_*i*_ for a given task instance starting at time *t*′ and target path Q^*^)		
**Strategy (*i* index)**	**Rule/Definition**	**Description**
*Random* *D*_*i*_(*t*) = {*p*|*p* ∈ *G*_*i*_(*t*)}		The random strategy does not consider learning, thus its domain of action is open to all BDT locations *p* at any time *t*.
$\begin{array}{cc} \begin{array}{c} Spatial\\ Generative\\ Discriminative \end{array} & \{\begin{array}{c} \;\\ \;D_{i}(t=t\prime )=\{p|p\notin R_{i}(t\prime )\vee p\in Q^{*}\}\\ \; \end{array} \end{array}$		At the begining of a given task, valid actions can be taken only in unexplored locations *p* or those belonging to the target path Q^*^.
$\begin{array}{cc} \begin{array}{l} Generative\\ Discriminative \end{array} & \{\begin{array}{l} \;\forall c|c\in R_{i}(t\prime )\wedge c\in Q^{*}\\ \;\to \text{subtree}(\text{neighbor}(c))\notin D_{i}(t\prime ) \end{array} \end{array}$		Additionaly, discard tree branches by using previous known *c* concep-icon relationships. subtree(*c*) is a function that yields *c* and the set of all locations below *c*, and neighbor(*p*) yields the neighbor of a given location/icon *p*.
**Domain of action updates (updates *D*_*i*_ while searching for correct feedback in path *Q*_*t*_ at time *t*)**		
$\begin{array}{cc} \begin{array}{l} Spatial\\ Generative\\ Discriminative \end{array} & \{\begin{array}{l} \;\forall p|\text{leaf}(p)\wedge p\in Q_{t}\to p\notin D_{i}(t+1)\\ \;\\ \;\forall p|p\in Q_{t}\wedge \text{neighbor}(p)\notin D_{i}(t)\\ \;\to p,\text{parent}(p)\notin D_{i}(t+1) \end{array} \end{array}$		The final selection of a path *Q*_*t*_ is always out of domain in the next iteration. leaf(*p*) is a function that is true for each BDT leaf (any 4th level location/icon).
		Bottom-up rule to discard parent nodes of *Q*_*t*_ when they have already being explore. parent(*p*) is a function that yields the location/icon in the level immediately above which leads to *p*.
Knowledge updates (updates *R*_*i*_ when learning the task instance of target path Q^*^ at time *t*)		
$Spatial\{\begin{array}{c} \forall p|\text{leaf}(p)\wedge p\in Q^{*}\to p\in R_{i}(t+1)\\ \forall p|p\in Q^{*}\wedge \text{neighbor}(p)\in R_{i}(t)\\ \to p,\text{parent}(p)\in R_{i}(t+1) \end{array}$		The final selection of a learned path Q^*^ is always in the learned set of upcoming tasks.
		Bottom-up rule to set parent nodes of Q^*^ as learned.
$\begin{array}{cc} \begin{array}{l} Generative\\ Discriminative \end{array} & \{\begin{array}{l} \;\forall c|c\in Q_{a}^{*}\cap Q_{b}^{*}\wedge c\notin \{Q_{j}|t_{a}^{*}<j<t_{b}^{\prime }\}\\ \;\wedge c\in \{Q_{j}|t_{b}^{\prime }\leq j\leq t\}\to c\in R_{i}(t+1) \end{array} \end{array}$		Concept *c* is considered learned if there are no selection mistakes between two tasks *a* and *b* which intersect in that concept. Task *a* is learned at time $t_{a}^{*}$, and task *b* starts at time $t_{b}^{\prime }$.

To track the BDT knowledge of the specific strategy *s*_*i*_, we define α_*i*_ as a measure of what still needs to be mapped (place or concept) of the BDT at a given time *t* :

(1) $$\begin{array}{r} \alpha_{i}(t)=1-\frac{\left|R_{i}(t)\right|}{\left|G_{i}(t)\right|} \end{array}$$

where *R*_*i*_(*t*) is the set of learned choices, *G*_*i*_(*t*) is the set of all distinct choices in the strategy's domain, and |·| is the number of elements of a given set. This parameter is the complement of the specific learning curve of each strategy. Specifically, α_*i*_ = 1 indicates complete lack of knowledge about the interface, and α_*i*_ = 0 indicates full knowledge.

Note that high-level strategies evolve according to the user's iterative interactions with the task. For instance, if the participant does not show evidence of learning any path, the learning curve of each strategy is a straight constant line at α_*i*_ = 1. When feedback becomes available (i.e., when the participant reaches the end of a path producing either a correct or incorrect answer), we ask each model how such observation changes or violates its expected probabilities regarding the nature of future feedback. As long as no learning is involved, all active models will answer equally to this query. However, as evidence of learning becomes available, each model will restrict the domain of possible future actions that are consistent with what the model predicts the participant's knowledge should be. A spatial model will label as a mistake any repetition of a path that previously gave positive feedback in the context of a different instruction. A generative model will label as mistakes actions that are inconsistent with a successful icon-concept mapping for which there is prior evidence. The discriminative model inherits the restrictions imposed by the generative model, but will also consider mistakes as those actions that do not take into account not-A type knowledge that the participant should have, given the history of feedback. An important consequence of the above is that strategies can yield the probability of clicking a given icon of the BDT without further training or modeling at any moment throughout the task.

It is worth noting that defining all possible strategies to solve the BDT is not necessary. To delimit the knowledge level of the participant, only the lower and higher bounds of the problem must be defined and more strategies in-between will only increase the framework's resolution. In the BDT case used here, the lower bound is necessarily the random strategy. The upper bound is set by the discriminative strategy because it is the best possible strategy to solve the BDT task (i.e., it requires the least exposure to positive feedback). Accordingly, we make the assumption that at any given moment, the user has all strategies at his disposal but that overt behavior is best captured by a weighted mix of them. We call this level the behavioral model of the framework.

#### 2.4.2. Behavioral modeling

Once the high-level strategies are defined, we turn to modeling the expectation about the use of a specific strategy or a combination of them by the participant. Such behavioral model is composed by a modular architecture of the four possible strategies, which interact between them in a HMM-like structure. This modular architecture has the advantage of modeling complex strategies as if they were a single abstract state in the behavioral model.

Formally, a HMM is defined by a finite set of hidden states, *s*_1_, *s*_2_, …, *s*_*n*_, and each time a relevant information arises (e.g., feedback) the system moves from one state *s*(*t*) = *s*_*i*_ to another *s*(*t* + 1) = *s*_*j*_ (possibly the same). The transition probabilities, *P*(*s*_*i*_ → *s*_*j*_), determine the probability of transiting between states: *P*(*s*_*i*_ → *s*_*j*_) = *P*(*s*(*t* + 1) = *s*_*j*_|*s*(*t*) = *s*_*i*_). The observable information of the process is a finite set of distinct observations, *v*_1_, *v*_2_, …, *v*_*m*_, and a probabilistic function of the states. Accordingly, each observation has an emission probability *P*(*v*_*k*_|*s*_*i*_), *k* ∈ {1, …, *m*}, of being seen under a state *s*_*i*_. As the system must start somewhere, it is necessary to define an initial probability distribution of the states: *w*_*i*_ = *P*(*s*(*t* = 0) = *s*_*i*_), *i* ∈ {1, …, *n*}. Given that the sets of hidden states and observations are defined a priori, the only values to estimate are the initial distribution and the transition and emission probabilities. Each strategy therefore takes the role of a hidden state at the behavioral level, which then yields the probability distribution of each observation for each trial.

##### 2.4.2.1. Emission probabilities

Although, the task can yield positive and negative feedback, an observer can interpret these observations in different ways depending on the situation. While positive feedback is unambiguous (a hit observation), negative feedback can have two different connotations: it is a mistake if, given previous actions, the observer is warranted to assume that the participant should have had the knowledge to avoid performing the action that produced such outcome. Observing such feedback will therefore mean evidence in favor of random behavior. Else, negative feedback is consistent with exploratory search behavior prior to the first positive feedback and, accordingly, not considered a mistake. The set of observations *V* is therefore defined as: *V* = {mistake, explore, hit}.

Since strategies are sensitive to the context, their emission probabilities change as the participant makes choices. At each sequence step, we calculate the emission probabilities within the subtree of possible future choices. Considering the last choice of the participant as the root of the subtree, we enumerate all possible future paths of actions *Q*, defining the following sets at step *t*:

(2) $$\begin{array}{r} H_{i}(t)=\{Q|(\forall a\in Q)[a\in D_{i}(t)]\wedge (\exists a\in Q)[a\notin Q^{*}]\} \end{array}$$

(3) $$\begin{array}{r} H_{i}^{*}(t)=\{Q|(\forall a\in Q)[a\in Q^{*}]\} \end{array}$$

where *a* represents a specific action needed to generate path *Q*, and Q^*^ the target path. *H*_*i*_(*t*) is the set of all paths that do not violate the strategy's rules, while simultaneously allowing the exploration of available choices. $H_{i}^{*}\left(t\right)$ is the set of paths that lead to positive feedback. Thus, emission probabilities for strategy *s*_*i*_ are defined as follows:

(4) $$\begin{array}{r} \begin{array}{l} P(V|s(t)=s_{i})\\ =\{\begin{array}{ll} \left\{0,\frac{\left|H_{i}(t)\right|}{\left|H_{i}(t)|+|H_{i}^{*}(t)\right|},\frac{\left|H_{i}^{*}(t)\right|}{\left|H_{i}(t)|+|H_{i}^{*}(t)\right|}\right\}, & \text{if }|H_{i}(t)|\;>0\vee |H_{i}^{*}(t)|\;>0\\ \{1,0,0\}, & \text{ otherwise} \end{array} \end{array} \end{array}$$

The first case represents emission probabilities for those strategies that, given their rules, allow for future exploration or exploitation. In the second case, when the rules of the strategy cannot explain the current actions, the emission probabilities are fixed to explain mistakes. This is also the case for the random strategy, which is assumed when the observer has no knowledge about the participant's strategy.

##### 2.4.2.2. Transition probabilities

It is possible, but not necessary, that a participant moves progressively through each of the increasingly complex strategies as he learns the structure of the task. Although, such progression may seem as discrete steps (i.e., first using a spatial strategy and then abandoning it altogether when conceptual knowledge becomes available), it is most likely that at any given moment of the task, the participant's strategy will fall somewhere in between, being better represented by a mix of strategy models. This is precisely the distribution captured by the behavioral model. To estimate it, it is necessary to model the interactions between strategies *s*_*i*_, in terms of transition probabilities and relative weights.

To obtain the transition probabilities we use a voting scheme based on emission probabilities, where each strategy distributes its own *P*(*V*|*s*_*i*_) depending on the ability of the strategy to explain a specific type of observation. Thus, for each observation *v* ∈ *V* we define the set of best explanatory strategies of *v* as follows:

(5) $$\begin{array}{r} B_{v}(t)=\{s_{i}|s_{i}\in \underset{s_{i}}{\text{arg max }}P(v|s_{i})\} \end{array}$$

Then, every step *t* in which the participant performs an action, transition links $l_{ij}^{t}$ between strategies *s*_*i*_ and *s*_*j*_ gain votes according to the following rules:

(6) $$\begin{array}{r} l_{ij}^{t}=\sum_{v\in V}P(v|s_{i})\{\begin{array}{ll} 1, & \text{if }s_{i}\in B_{v}(t)\wedge i=j\\ \frac{1}{\left|B_{v}(t)\right|}, & \text{if }s_{j}\in B_{v}(t)\wedge s_{i}\notin B_{v}(t)\\ 0, & \text{otherwise} \end{array} \end{array}$$

These rules represent three cases: (a) Any strategy that belongs to *B*_*v*_(*t*) strengthens its self-link, not sharing its *P*(*v*|*s*_*i*_) with other strategies. (b) Strategies that do not belong to *B*_*v*_(*t*) generate links to those that best explain the observation *v*, losing their *P*(*v*|*s*_*i*_) in equal parts to those that best explain *v*. (c) Strategies that do not explain *v* or do not belong to *B*_*v*_(*t*) do not receive votes for observing *v*.

In the case of the random strategy, its emission probabilities are fixed to {1, 0, 0}, therefore the above rules do not generate links with other strategies in the case of exploring or exploiting the BDT knowledge. In order to overcome this limitation, we define the set of strategies that can leave the random strategy as those that do not see the current action as a mistake, plus the random strategy itself:

(7) $$\begin{array}{r} U(t)=\{s_{i}|P(\text{mistake}|s_{i})=0\}\cup \{s_{random}\} \end{array}$$

Then, the links from random strategy are voted as:

(8) $$\begin{array}{r} l_{random\;j}^{t}=\frac{1}{\left|U(t)\right|}\{\begin{array}{ll} 1, & \text{if }s_{j}\in U(t)\\ 0, & \text{otherwise} \end{array} \end{array}$$

Note that $\underset{v}{\sum }P\left(v|s_{i}\right)=1$ for each strategy, thus the vote sharing scheme is always normalized.

The value of these links represent only what happens at the current time. The participant's actions, however, can be tracked historically by defining an observation window of the process τ, which modulates the weight of the votes over time. Therefore, at time *t*, the amount of cumulative votes *L* between strategies *i* and *j* is defined by:

(9) $$\begin{array}{r} L_{ij}(t)=\frac{\sum_{n=0}^{t}l_{ij}^{q}\tau (n)}{\sum_{n=0}^{t}\tau (n)}=P(s_{i}\to s_{j}) \end{array}$$

Here we define τ as a Gaussian function with a standard deviation of σ steps, normalized to a maximum of 1 at the current time *t*:

(10) $$\begin{array}{r} \tau (n)=e^{\frac{-{\left(n-t\right)}^{2}}{2\sigma^{2}}} \end{array}$$

The result is a set of directed interactions (i.e., transition probabilities) among different strategies, storing historical information of the participants' behavior.

##### 2.4.2.3. Weights optimization

Weights represent the probability distribution across strategy models at each iteration. Once emission and transition probabilities are known, weights are updated by comparing the cost, in terms of probabilities, to start in some strategy and end in the best transition link that explains an observation type. This allows us to compare the transition that best represents the current state of the behavioral model (to where the system is moving) for a specific observation, and how much it costs for each model to reach that point.

The cost, in terms of probability of moving from the state *s*_*k*_ to *s*_*j*_ (which could be the same), given an observation *v* ∈ *V*, is represented by *P*(*s*_*k*_ → *s*_*j*_)*P*(*v*|*s*_*j*_). Note that there may be more than one best transition for the system. Accordingly, we define set of best transition links as:

(11) $$\begin{array}{r} \left\{(s_{k},s_{j})|\underset{s_{k},s_{j}\in M}{\text{arg max }}P(s_{k}\to s_{j})P(v|s_{j})\right\} \end{array}$$

where *M* represents the set of all strategy models. Equation (11) implies that the behavioral model identifies the transition from *s*_*k*_ to *s*_*j*_ as one of the most representative in case of observing *v* at time *t*. In case more than one best transition exists, the most convenient path is optimized. Then, for an initial state *s*_*i*_, the cost of reaching the best transition link is defined as the path that maximizes the following probability:

(12) $$\begin{array}{r} L_{ii}(t)P(v|s_{i})P(s_{a}\to s_{b})P(v|s_{b})\cdots P(s_{k}\to s_{j})P(v|s_{j}) \end{array}$$

We use *L*_*ii*_ as the prior for the initial probability *w*_*i*_(*t*), so that models with *w*_*i*_(*t* − 1) = 0 can be incorporated in the optimization at time *t*. Note that Equation (12) is equivalent to the Viterbi path constrained to a fixed observation *v*.

Then, the weight of strategy *s*_*i*_ at time *t* + 1 is represented by the total cost for the set of observations *V*:

(13) $$\begin{array}{r} w_{i}(t+1)=\sum_{v\in \;V}w_{i}(t)P(v|s_{i})\cdots P(s_{k}\to s_{j})P(v|s_{j})\tau_{v}(t) \end{array}$$

where τ_*v*_(*t*) yields the relevance of observation *v* in the total cost function, given an observation window τ (such as defined in Equation 10):

(14) $$\begin{array}{r} \tau_{v}(t)=\sum_{n=0}^{t}\tau (n)\{\begin{array}{ll} 1, & \text{if }v(t=n)=v\\ 0, & \text{otherwise} \end{array} \end{array}$$

The most likely observation at time step *v*(*t*) is defined by consensus regarding the observation with the best average emission probabilities:

(15) $$\begin{array}{r} v(t)=\underset{v\in \;V}{\text{arg max}}\sum_{s_{i}\in M}P(v|s_{i}) \end{array}$$

Finally, in order to obtain a probability distribution over the search strategies, each weight is normalized by the coefficient *W* defined as:

(16) $$\begin{array}{r} W=\sum_{i\in M}w_{i}(t+1) \end{array}$$

Note that if two or more models have the same domain of action (e.g., both concept-based strategies have the same domain of action for target path Q^*^), we consider only the one with greatest knowledge. Otherwise the normalization is unfair to the other models.

##### 2.4.2.4. Learning curve

Each strategy's individual knowledge of the BDT, α_*i*_, can be combined with its respective weight *w*_*i*_ to produce a mixture of basal strategies α^*^. This is accomplished by a weighted sum:

(17) $$\begin{array}{r} \alpha^{*}(t)=\sum_{i\in M}\alpha_{i}(t)w_{i}(t) \end{array}$$

Recall that α_*i*_ is the complement of the specific learning curve of each strategy. Therefore, the approximate learning curve of a given participant can be obtained as: 1 − α^*^.

##### 2.4.2.5. Predicting participants' choices

As the models' weights are known previous to icon selection, we can build a Mixture of Experts (Jacobs et al., 1991) where search strategy models become the experts that must answer the question “Which icon is the participant most likely to click in the next step?”. Because each model can produce any of two possible actions, i.e., clicking on the left or the right icon, the most probable next choice at step *t* will be the one that has the largest support as expressed by the weighted sum rule of the ensemble:

(18) $$\begin{array}{r} \underset{a\;\in \{\text{left},\text{right}\}}{\text{arg max}}\mu_{a}(t)=\sum_{i\in M}w_{i}(t)\{\begin{array}{ll} 1, & \text{if }a\in h_{i}(t)\\ 0, & \text{otherwise} \end{array} \end{array}$$

where *h*_*i*_(*t*) represents the expert-prediction of the strategy *i*. Prediction can be based on dichotomous decisions or random selection. Dichotomous decisions occur when there is only one possible action in the strategy's domain of action so that it will be selected with probability equal to 1. Alternatively, when both possible actions belongs to the strategy's domain (i.e., have equal probability of being selected) or when neither of the actions belongs to the domain (i.e., the strategy is in the inactive set), a coin is tossed to choose at random (50/50 guess).

It is worth noting that the prediction capability of each strategy depends on the size of its domain of action. Strategies with wider exploratory behaviors often have larger domains, and consequently, less predictive power due to the number of paths that can be selected. This aspect is captured by the number of 50/50 guesses, because, as mentioned above, whenever the strategy has more than one equally likely possibility of action, it must choose at random. This does not mean that the strategy itself is failing to capture the participants' choice of action, but that the conditions are ambiguous enough to keep looking for the correct path.

Finally, to better visualize the search process of participants (and groups of participants), we introduce three scores. As any sequence of choices can be the consequence of different degrees of expertise (from fully random behavior to goal-directed exploitation), we define a scale where we assign points depending on the most likely observation *v*(*t*) that yields the behavioral modeling (Equation 15) at each step of a sequence of choices *Q*.

To measure the degree of expertise for path *Q*, realized between time steps *t*_*a*_ and *t*_*b*_, we define the expertise score as:

(19) $$\begin{array}{r} \text{expertise}=\frac{1}{\left|Q\right|}\sum_{t_{a}<=t<=t_{b}}\{\begin{array}{ll} 1, & \text{if }v(t)=\text{hit}\\ 0.5 & \text{if }v(t)=\text{explore}\\ 0, & \text{if }v(t)=\text{mistake} \end{array} \end{array}$$

where expertise equal to 1 means exploitation behavior and expertise equal to 0 means completely random behavior. Values in-between represent various degrees of exploration.

If only quantifying exploration and exploitation rates, we assign points equal to 1 only if *v*(*t*) match the explore/hit observation respectively:

(20) $$\begin{array}{r} \text{exploration}=\frac{1}{\left|Q\right|}\sum_{t_{a}<=t<=t_{b}}\{\begin{array}{ll} 1, & \text{if }v(t)=\text{explore}\\ 0, & \text{otherwise} \end{array} \end{array}$$

Likewise, exploitation score is defined as:

(21) $$\begin{array}{r} \text{exploitation}=\frac{1}{\left|Q\right|}\sum_{t_{a}<=t<=t_{b}}\{\begin{array}{ll} 1, & \text{if }v(t)=\text{hit}\\ 0, & \text{otherwise} \end{array} \end{array}$$

## 3. Results

We present the results organized around four main themes: behavioral results, model parameter dependence, individual differences in the learning process, and prediction of participants' choices.

### 3.1. Behavioral results

**Figure 2:**
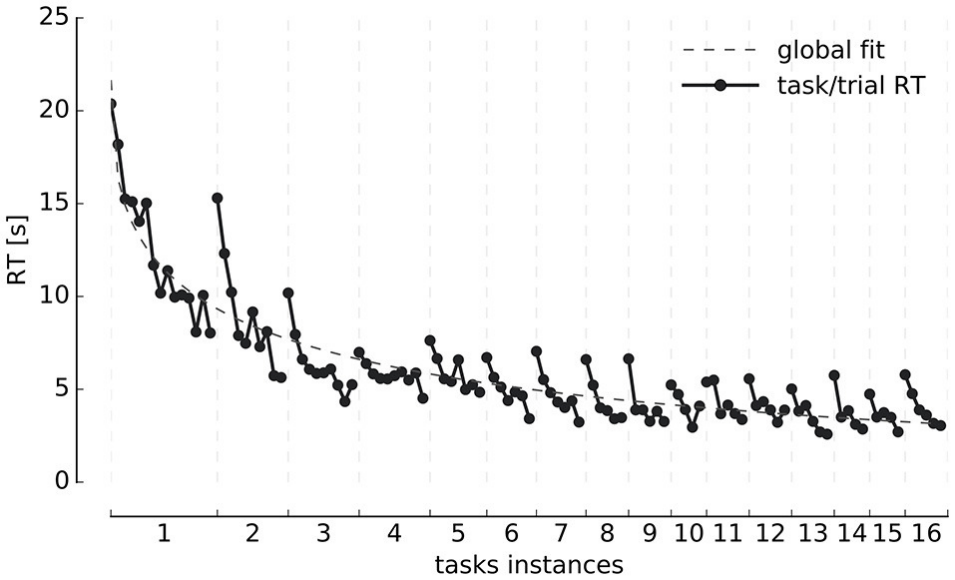
Reaction times. Evolution of trial reaction times (y-axis) grouped by tasks instances (x-axis) and averaged over all participants. The global RT curve fit, corresponds to an exponential decay function of the type: $\lambda_{1}\exp\left(-\lambda_{2}x^{\lambda_{3}}\right)$, where λ_1_ = 21.6 is the starting average RT, λ_2_ = 0.28, and λ_3_ = 0.4. The coefficient λ_3_ is necessary since the drop in time is not as steep as when λ_3_ = 1 (the usual exponential decay constant).

Reaction times (RT) averaged over all participants for individual repetitions of each task instance and for the overall experiment are presented in Figure 2. As different participants have different number of trials per task instance, we calculated a representative average of each instance through the following interpolation scheme: we estimated a bin size for each task by calculating the average number of trials per task over the group of participants. Each individual's task-related vector was then linearly interpolated in that common space. This preserves the relative time that it takes—on average—for the participants to complete each task instance, while it allows to visualize the average of the reaction time and learning curves (**Figure 4**) more naturally.

A consistent two-fold exponential structure is visible revealing the development of expertise. Each individual task instance (same instructions) takes progressively less time to solve as participants repeat it. Likewise, successive instances of the task (different instructions) take progressively less time to solve as the session unfolds. Participants take an average of 29 ± 9 min (Mean ± *SD*) to complete the entire experimental session, with a minimum of 13 min and a maximum of 40 min, which was the maximum time allowed to solve the task.

### 3.2. Model parameter dependence

**Figure 3:**
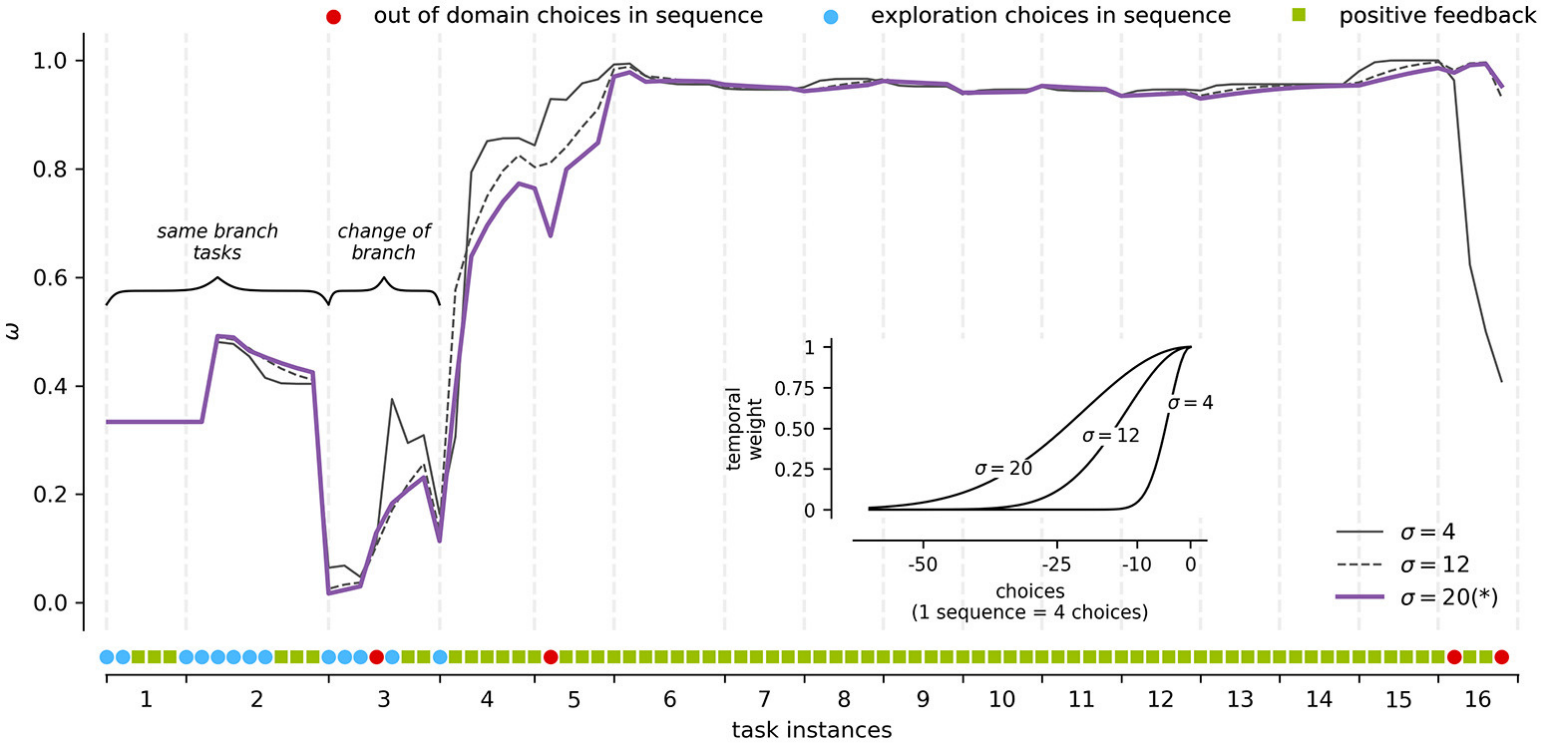
Framework parameters modulation. An example for a participant's discriminative strategy weight (y-axis) for each task instance (x-axis) at different τ values (see inset). The inset x-axis presents the distance in choices from the current time and the y-axis the respective kernel weight. The curve marked by (^*^) represents the σ used for modeling the rest of results presented in the section.

To illustrate how the modeling results are influenced by the observation window of the process τ (Equation 10), we analyze the dynamics of τ while tracking the weight of a participant's discriminative strategy (Figure 3).

The observation window τ affects how observations and the voting scheme impact the point of view of the observer when determining the use of a given strategy by the user. This parameter directly affects the transition probabilities of the behavioral modeling, and the weights through the temporal relevance of the observations. To test its sensitivity, we built Gaussian kernels of σ equal to 1, 12, and 20 steps, which represent 1, 3, and 5 trials/sequences of choices (see inset in Figure 3 for a graphical representation of the kernels). These kernels cover cases between the maximum temporal weight in the current choice (σ = 1) to 50% of the temporal weight at approximately 5 trials of distance (σ = 20). The highest σ value was chosen such that it includes the temporary extension of the average number of trials to find positive feedback (8 paths), when the domain is the entire BDT.

As seen in Figure 3, these cases capture the general dynamics of the learning process. For example, task instances 1 and 2 are statements which belong to the same branch of the BDT. The participant uses the information learned in instance 1 to answer instance 2, what is reflected positively in the weight of concept-based strategies such as the discriminative strategy. Conversely, task instance 3 does not belong to the same branch of instances 1 and 2. This time the participant is faced with a more explorative situation, which is reflected in a decrease in the weight of the discriminative strategy. Finally, around task instances 4 and 5 the weight of the strategy is consolidated around the maximum weight, which is expected in a fully discriminative behavior, where 4 paths of positive feedback can describe the full knowledge of the BDT.

Regarding the specific sensitivity of τ across kernels, small kernels tend to react faster to changes in the style of the participant, punishing or rewarding the weight of a strategy very quickly. For example, a σ = 1 kernel produces “spikes” in Figure 3 in tasks instance 3 when the participant goes from mistake to exploration (rewarding case). Likewise, in tasks instance 16, when participant performs incorrect actions in a context of full knowledge, the weight of the strategy is punished abruptly to match other strategies that are compatible with such behavior. On the contrary, large kernels tend to produce smoother transitions, as a more extensive history is taken into account when calculating the relevance of the current observation. For example, task instance 4 in Figure 3 represents a shift in the strategies' weights toward discriminative behavior. As expected, the σ = 1 kernel has a steeper curve than the σ = 20 kernel around shift time. Finally, when a strategy is irrelevant in the current weights distribution, as seen from task instance 6 to 15 of Figure 3, distinct kernels make no difference in the strategy's final weight curve.

Although, τ can be set for different usage scenarios and types of tasks, we use τ(σ) = 20 for modeling our data hereafter. This choice produces smoother changes in the α curves, clearly showing the process of expertise acquisition.

### 3.3. Individual differences

**Figure 4:**
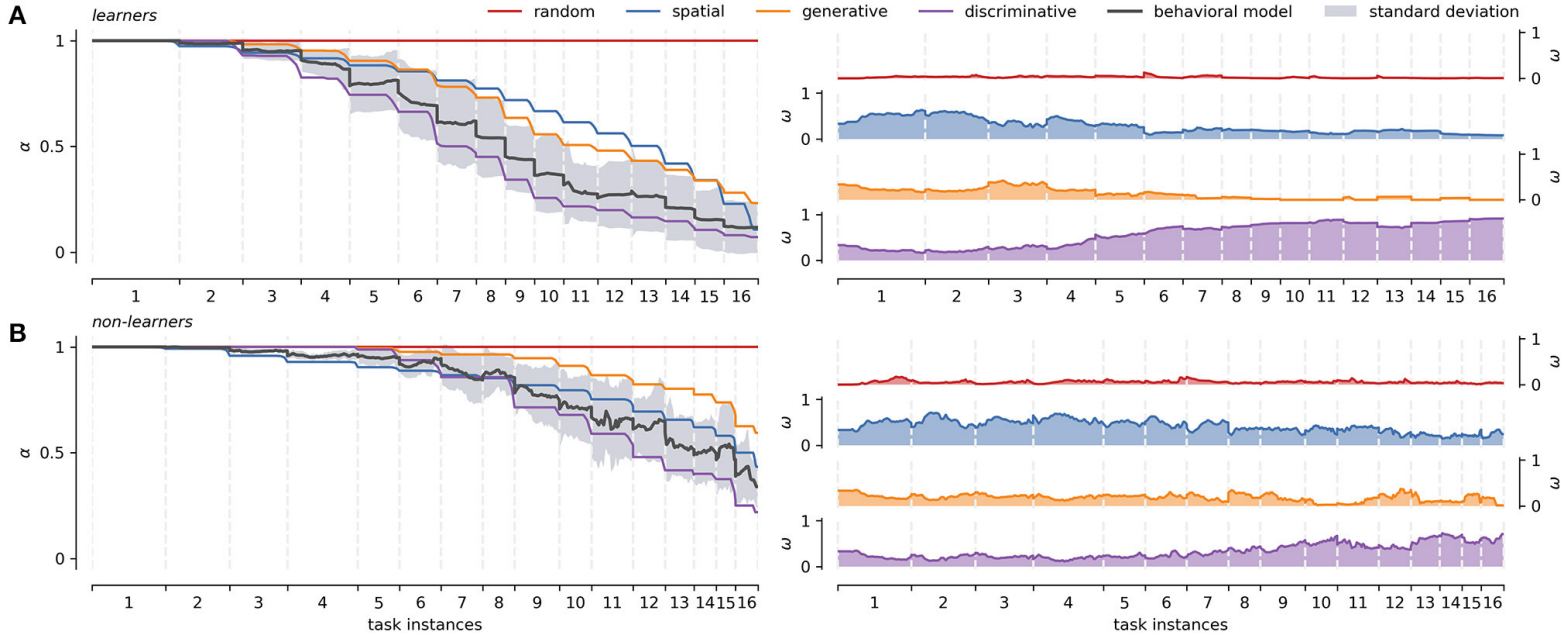
Learners and non-learners average strategies across tasks. **(A)** Right panel: characterization of the strategy models averaged over all participants (66.7%) that managed to solve the sixteen different instances of the task (x-axis). Left panel: average weights of each strategy models over the same group of participants. **(B)** Right panel: characterization of the strategy models averaged over all participants (33.3%) that didn't fully learn the task. Left panel: average weights of each strategy models over the same group of participants. The behavioral modeling represents the averaged individual approximation (basal strategies weighting) and is shown with its standard error across tasks. Dispersion of α curves for basal strategies is not presented to facilitate visualization. The latter can be seen in greater detail in Figure 5 below. The same interpolation scheme as in Figure 2 is used.

It was possible to distinguish two groups of participants according to whether they managed to solve the task (learners, *N* = 14) or not (non-learners, *N* = 8) in the allowed time window (40 min). We consider learners (Figure 4A), all participants who managed to complete the 16 tasks instances, reaching full knowledge of the interface as evidenced by consistent positive feedback. Conversely, the non-learners group (Figure 4B), is composed by participants who did not complete the task within the time limit, or failed to reach full knowledge of the interface.

For the learners group, the left panel in Figure 4A shows the distinctive shape of each strategy as they reach full knowledge. The right panel in Figure 4A shows the average weight of each strategy model across the 16 different task instances. Recall that the absence of learning under the random strategy is represented as a straight, constant line at α = 1. In the spatial model, as learning progresses, the number of attempts necessary to encounter positive feedback decreases exponentially. Finally, generative and discriminative models are represented by increasingly steep sigmoid functions, as complete knowledge of the BDT can be obtained with less positive feedback exposure. It is worth noting that the definition of learning for concept-based strategies (no selection mistakes between two consecutive paths with the same target concept), causes that not all learning curves reach zero for all participants. This is mainly because not all concepts can be checked for the learning condition in one run of the task. The average group-level behavioral modeling remains between the space delimited by the generative and discriminative strategies, following more closely the discriminative strategy. The right panel in Figure 4A shows that the weight of the discriminative strategy starts outperforming the rest of the strategies around task instance 4. Before that, the spatial strategy is used to locate positive feedback. Generative strategy has a short transition period between spatial and discriminative strategies around task 3, loosing prominence quickly after that.

In contrast, non-learners tend to go through successive task instances without obtaining positive feedback (i.e., they are prompted to continue after persistent mistakes, see Methods Section). Accordingly, in this group all strategies suffer substantially regarding knowledge completion, never reaching full knowledge (Figure 4B left panel). In general, non-learners do not generate as much explicit knowledge as learners do, focusing on spatial exploration over conceptual mapping in order to find positive feedback. This is reflected in the fact that the spatial α curve surpasses the generative α curve. Importantly, however, some implicit knowledge seems to be generated based on the tasks that they do manage to complete. This would explain why the discriminative strategy gains weight toward the end of the task (Figure 4B right panel task instance 9). In this group, the average behavioral modeling moves between spatial and discriminative strategies.

**Figure 5:**
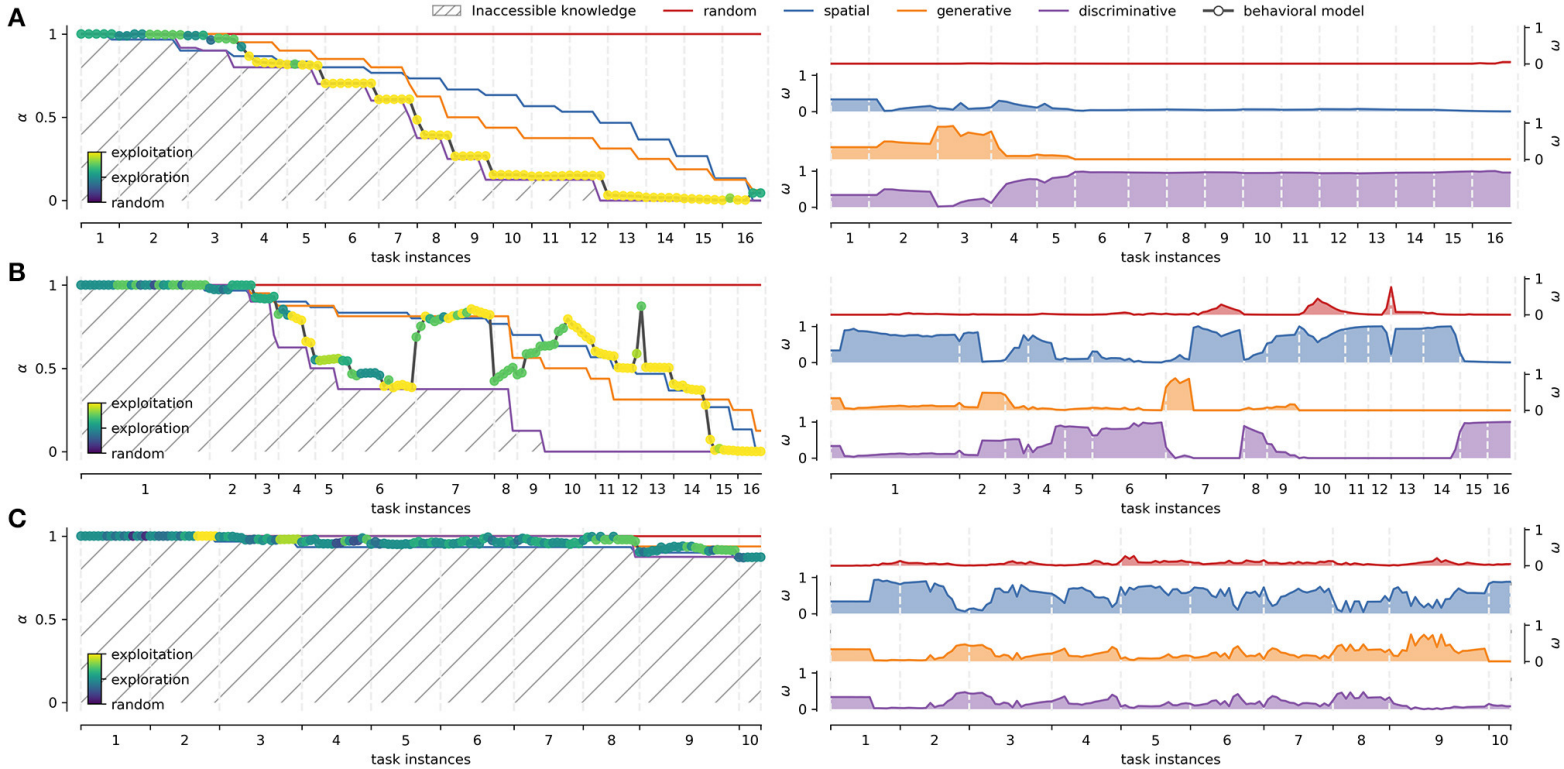
Study cases. Three representative individual cases according to task performance. **(A)** Highly proficient learner. **(B)** Less proficient learner. **(C)** Non-learner. The left panel of each case shows the deployment of basal strategies in terms of α during the overall duration of the experiment (x-axis). Colored dots represent different degrees of expertise used to explore/exploit the interface knowledge (Equation 19). The right panels of each case shows the evolution of the corresponding weights for each strategy across task instances. The average of the basal strategy weights yield α^*^ for the behavioral modeling.

Figure 5 presents the deployment of strategies and their corresponding weights for representative cases of the two groups. In these cases, each strategy reaches different levels of knowledge depending on the participants' context. A highly proficient learner, Figure 5A, usually goes from random to discriminative strategies progressively, as seen in the weights panel. Few mistakes are committed, and the learning curve (1−α^*^) approximates a sigmoidal shape. A less proficient learner such as the one shown in Figure 5B, initially goes through a similar process, but also through periods of difficulty in solving the task (e.g., from task instances 7 to 9 in Figure 5B). The corresponding learning curve presents a sigmoid shape interrupted by intervals of evidence for spatial and random strategies, which is caused by explorations and mistakes. These intervals have different durations and extensions, finally disappearing when the discriminative strategy regains dominance. A non-learner, as the case shown in Figure 5C, typically has a similar starting strategy distribution as learners. However, instead of converging to the discriminative strategy, the participant falls back mainly to the spatial strategy. As they do not appear to learn most of the concepts, they must search for positive feedback in less efficient ways during the whole task.

### 3.4. Predicting participants' choices

**Figure 6:**
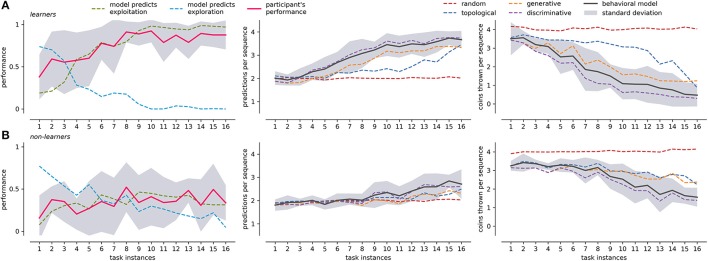
Participants performance, framework prediction, and 50/50 guesses averaged across tasks. **(A)** Presents results for the learners group: the leftmost panel shows the participant's averaged performance (y-axis), compared with exploration/exploitation performance as predicted by the behavioral modeling; the center panel present the average number of predicted choices (y-axis) in a sequence for behavioral model and individual strategies; the rightmost panel present the number of 50/50 guesses (y-axis left) for center panel. The x-axis for all panels is the average across the 16 different tasks. **(B)** Presents the equivalent measures of **(A)** for non-learners. The corresponding standard deviation of participants' performance and behavioral modeling is shown in gray. Standard deviation for the rest of the curves is not presented to facilitate visualization.

In order to better understand the actions of the participants, we tracked the emission probabilities of exploration (Equation 20) and exploitation (Equation 21), comparing them with the actual performance of the participant in the task. Figure 6 leftmost panel shows these comparisons for learners and non-learners. In the case of learners, the model identifies complementary curves for exploration and exploitation (exploration + exploitation ≈ 1). Learners tend to be more explorative at first, but their performance falls between exploration and exploitation. From task instance 4 onwards, the performance of learners is similar to what the model predicts as exploitation. After that, exploration behavior tends to disappear and exploitation increases. This is congruent with Figure 4A left panel, where learners begin to use the discriminative strategy more consistently from task instance 4. In contrast, non-leaners do not have complementary exploration and exploitation curves. This is because of the random behavior during the search of positive feedback. Although the actual behavior of non-learners is similar to the exploitation curve, the performance level remains under 50%. This is probably because they only map the easiest levels of the BDT (level 1 and 4), while exploring the rest of the tree in each task instance.

To compare the performance of our behavioral model, we use a Mixture of Experts (Equation 18) to predict the participant's most likely next choice. Prediction outputs two values: a next possible action and the number of 50/50 guesses (see Section Methods), normalized by the experts' weights. The same concepts apply to individual strategies, so that the prediction capabilities can be compared by the prediction output of each strategy. Figure 6 center and rightmost panels compare choice prediction capabilities and the number of 50/50 guesses for both learners and non-leaners. At the beginning, when the knowledge of the BDT is still poor, we expect a high 50/50 guess rate with a consequently low prediction accuracy. In both groups, prediction accuracy starts at around 25% (two choices). This is because, on average, it is not difficult to guess the first and four level icons at random, regardless of it being a participant or an observer. From then on, learners progressively improve their performance, reaching values close to 90% halfway through the experiment, and near 100% toward the end of the task. The number of 50/50 guesses follows the previous trend, stabilizing near zero around task instance 10. In the case of non learners, the number of 50/50 guesses is quite high, only decreasing around task instance 9 and converging to two 50/50 guesses choices. The initial prediction capabilities of two choices is maintained throughout the course of the task, and only rises to about three choices toward the end of the task.

In both groups the leading single strategy is the discriminative, which is followed closely by the behavioral model. This is consistent with the results presented in Figure 4, where the weight of the discriminative strategy follows the critical moments where it differs from the rest of the strategies (task instance 4 for learners and 9 for non-learners). This suggests that the core rules of the BDT mapping are learned through this mechanism. However, the discriminative strategy is not able to fully explain the behavior of the participants in all the stages of expertise acquisition (and the other strategies do not explain it either). This may be due to the fact that before the critical point of the discriminative strategy, participants are not as systematic as the strategies propose (or it is necessary to define different search strategies to explain such behavior). In the case of learners, the behavioral model predicts more exploratory behavior (Figure 6 leftmost panel) before that point. In other words, the modeling framework predicts that there will be a greater amount of exploration, independent of the exact way in which it would take place.

## 4. Discussion

We have presented here a modeling framework which, on the basis of stereotyped, well-defined search strategies, is able to track the development of expertise in a sequential decision-making task. We have tested the framework using a novel BDT task that has the potential to provide a well-defined computer interface scenario for the study of expertise acquisition in real world situations. By following individual learning processes, when confronted with users that are able to learn the task, our model quickly reaches a point where it can predict the users' most likely next choice. This suggest that the framework presented here could play a role in the development of adaptive interfaces where sequential choices are required to reach a desired outcome.

We organize the following discussion around four main issues: the modeling framework choice and its consequences for both the tracking of the learning process and for adaptive interface design; the structure of the sequential decision making task; the criteria for temporal and spatial information encoding; and the issue of individual differences among users.

### 4.1. Modeling framework

As outlined in the introduction, a host of approaches exist to deal with the problem of modeling decision making behavior in general (Sukthankar et al., 2014), and sequential decision making in particular (Walsh and Anderson, 2014). Here we have chosen to follow an HMM-based approach that, in line with type-based methods, relies on a set of pre-defined strategies to model a potentially infinite set of behaviors (Albrecht et al., 2016). This approach reduces the dimensionality of the problem while at the same time aims to provide some insight into the learning and expertise acquisition process.

While the approach presented here is indeed inspired by the HMM structure, we do not use traditional algorithms to determine the parameters of the HMM (e.g., BaumWelch, see Rabiner, 1989 for more details). This is because, in our task, emission and transition probabilities are dynamic consequences of the participant's actions. Therefore, we can ask the strategies for the emission probabilities, because they directly operationalize possible outcomes (as a consequence of their formal definition). Transition probabilities are, in contrast, a more open problem because they define how the HMM is connected (i.e., its structure), which can itself change as learning happens. To deal with this issue, we define transition probabilities in terms of the comparative changes of emission probabilities between strategies. This is because, as discussed above, strategies formalize actual domains of action so that their comparison allows us to determine toward which strategy it is better to transit in order to best explain the current choice made by the participant. Finally, in the case of weight optimization, we use a formulation which is equivalent to finding the Viterbi path in a HMM (also described in Rabiner, 1989).

As our results show, the modeling approach presented here was able to consistently follow the performance of individual users as they became familiarized with the task. In those that were able to discover the underlying icon-concept mapping and fully solve the task (learners), the model was able to match their performance as early as the fourth instance of the task. More importantly for the question of how learning proceeds in a sequential decision making situation, the use of pre-defined strategies suggests that learners and non-learners are likely to use different approaches to the problem: while learners dwell on spatial, brute-search behavior only during a short while and then rapidly start using concept-mapping knowledge (mostly in discriminative terms), non-learners persist in spatial searches and only well into the task do they start showing behavior of consistent icon-concept mappings.

However, to the extent that the pre-defined strategies represent one possible set of high-level behaviors among many, our approach cannot provide definite evidence that users actually use such strategies. In this sense, it may well be the case that a different modeling approach (or a different subset of strategies) might capture equally well the user's behavior. This is, of course, a pervasive problem in behavioral modeling when users are free to tackle the problem at hand in whatever way they choose. Approaches that combine modeling with subjective reports such as Mariano et al. (2015) are therefore an interesting extension and worth exploring further. Indeed, Beta Process Hidden Markov Models (BP-HMM) (Fox et al., 2009) such as the one used by Mariano et al. (2015), consider libraries of states. Here, one pattern of states, represented by an HMM, is active at current time. This approach captures the idea of multiple patters of HMM structures, but the interpretation of such patterns in terms of the user's behavior is necessarily an exercise that has to be done a posteriori. Nevertheless, the pre-defined strategies that we chose here are informed by the structure of the problem and represent increasingly efficient ways to solve it, thus representing a valid alternative in the context of type-based approaches while being more sensitive to potential differences in the way users approach the task. Other modeling techniques, such as Interactive Partially Observable Markov Decision Processes (I-POMDP) (Gmytrasiewicz and Doshi, 2005), also rely on a similar approach by using predefined intuitions to solve the task (i.e., types of behaviors/strategies), thus allowing to capture the learning of users more efficiently and faster.

A final point regarding the use of a subset of high-level behaviors is worth noting: for instance, as shown in Figure 6 it could be argued that considering the discriminative strategy is enough to yield comparable results in terms of tracking the behavior of those who learn the task. This is expected given that by the fourth iteration, users should have been exposed to most of the discriminative knowledge necessary to fully map de problem. Yet, if we chose this approach, we would have no insight into potentially relevant strategies that unfold throughout the learning processes.

In addition to the previous considerations, an a posteriori validation of the approach is its capacity to predict the behavior of individual users. When it comes to studying the agent's interaction with a user, prediction inference is usually performed over the users' goals (Oh et al., 2011; Ramírez and Geffner, 2011). Such models identify the goal and the necessary output actions to fulfill that specific goal. In contrast, we specify the goal and our inference is about the way in which the user perform the actions in a search process. The strategies defined here represent policies of actions that could be optimal during different stages of learning. In this sense, prediction performance is not in and by itself, the primary measure of the model's capacity. For instance, even with poor prediction performance (e.g., Figure 6B center panel), the behavioral model can follow the actual performance of the participants based on the exploitation score. In addition, the number of 50/50 guesses can be considered an estimation of how good the prediction could be. As such, this approach (and extensions of it in terms, for instance, of a different set of strategies) could represent a viable approach to inform the construction of flexible interfaces that can adapt to the different moments of the learning curve of their users.

### 4.2. Task structure

There are several types of sequential tasks studied in the literature (Ruh et al., 2010; Diuk et al., 2013; Friedel et al., 2014; Huys et al., 2015). In this context, decision trees present an interesting scenario, because they naturally embody the most basic structures of a sequential decision-making situation (Fu and Anderson, 2006) while allowing for a clear description of the task structure. Here we chose a BDT design to instantiates a simple case of sequential action in Human-Computer Interactions (HCI).

An important aspect of our BDT design is the feedback structure. Specifically, participants have to reach the end of each branch before obtaining information about the appropriateness of previous decisions. In this kind of limited feedback scenario, a *credit assignment problem* appears (Fu and Anderson, 2008; Walsh and Anderson, 2011, 2014). This means that participants have to learn how to use the consequences of their actions to assign value to different parts of the sequential choice. In our task, participants face two main types of credit assignment problems: on the one hand, negative feedback is not enough in and by itself to discover at which point of the BDT wrong decisions were made. On the other hand, positive feedback can only be used by the participant to generate specific icons-concepts mappings through successive exposures to different instances of the task.

One could argue that immediate feedback interactions (such as interfaces characterized by labeled icons or explicit icon-concept mappings) is the dominant mode of HCI interaction and should therefore be the target for behavioral modeling. This would have the advantage of sidestepping the credit assignment problem. However, limited feedback scenarios such as the one used here have the advantage of making the task more difficult (Walsh and Anderson, 2011), therefore revealing the learning process more clearly. This has obvious advantages in terms of making the process of expertise acquisition by different users explicit and thus available for modeling efforts. Moreover, given the nature of our behavioral model, which deals with relationships among strategies, immediate feedback scenarios could be considered as a particular case for the framework. When interfaces are built on the basis of labeled icons, only two strategies are required: spatial and discriminative: the spatial strategy is necessary to map the physical layout and arrangement of information throughout the interface; the discriminative strategy, on the other hand, allows one to relate complementary or neighboring icons to future (alternative) task instances. The generative strategy, in contrast, is unnecessary because the primary icon-concept mapping is explicit from the start.

### 4.3. Temporal and spatial encoding criteria

Related to the credit assignment problem, another important issue arises when modeling behavior, especially in sequential decision making situations. This issue, which has received much less attention from modeling studies, pertains the criteria that one sets in order to determine which observations count as relevant information (Behrens et al., 2007). Such criteria can be of temporal nature (for instance, how much of past experience we consider when planning future actions) or spatial (for example, how much of the BDT's structure is remembered and used for making decisions).

In our work, the function-parameter τ determines the influence of past outcomes on the behavioral modeling. Here we have chosen a continuous Gaussian kernel (Equation 10) with a peak at the current time. This choice aims to capture underlying short term memory processes whereby current items have a higher probability of influencing behavior than those encountered previously (Cowan, 2008). Whether by interference of novel task information or due to temporal decay, current information cannot not persist indefinitely and therefore it is necessary to consider the dynamics of its causal influence on ongoing decision making (Lewandowsky and Oberauer, 2009; Barrouillet et al., 2012). The choice of the Gaussian kernel could be a target for improvement, eventually considering the possibility of adapting it to individual users' profiles. Nevertheless, similar exponential metrics, including Gaussian kernels have been used to model complex memory processes and therefore represents a viable first choice (Brown et al., 2007).

In addition to the temporal problem, a spatial navigation problem arises that is related to the localization of feedback (Madl et al., 2015). To localize the correct path, as well as to propagate credit to previous choices, it is necessary for the model to encode some type of memory of the user's actions (Fu and Anderson, 2008). In our work, each strategy has two types of rules: a global memory associated to learning the overall BDT structure according to that particular strategy across task instances, and a local memory, which is associated with narrowing the domain of action leading to correct feedback in the current instance of the task. Importantly, such encoding does not forget landmarks that have been encountered by the participant and in this sense does not represent actual memory processes. However, this is in principle not necessary because we are dealing with a highly restricted situation in which a full mapping of the task can happen within one experimental session. Indeed, we use a task repetition criterion to ensure learning and make forgetting more difficult (Corazzini et al., 2008). Eventually, when dealing with more complex search scenarios, taking into account that the user might forget a previously encountered location might be necessary (Baumann et al., 2011; Liverence and Scholl, 2015). Nevertheless, we account for apparent reductions in the user's knowledge of the BDT structure using the random model to penalize actions that, according to the model's encoding of the user's previous actions, represent mistakes in landmark choices.

### 4.4. Individual differences among users

One of our main results, which is related to the structure of the task, is the notorious difference among participants in terms of performance. Beyond the evident interest this has in terms of underlying psychological and brain mechanisms, from our perspective, this represents an opportunity to contrast the performance of participants of different skill levels when dealing with search problems (Sacchi and Burigo, 2008). Our task reveals that a standard group of participant is not uniform and can be separated in learners and non-learners, as a first level of analysis. These two groups differ not only in terms of whether they manage to complete the task, but also in terms of which strategies they prefer.

Such differentiation is relevant when we want to distinguish experts from non-experts. When dealing with a problem-solving situation, experts and non-experts differ on a number of dimensions. For instance, they differ in how they search for information (Barrick and Spilker, 2003; Hershler and Hochstein, 2009), make decisions (Connors et al., 2011; Gorman et al., 2015) or pay attention (Schriver et al., 2008), among others. Taking into account these differences is a critical step toward a more natural and truly adaptive HCI, as the user's needs could be focused more accurately according to their level of expertise. For example, being able to identify the strategy that is currently being deployed could enable the interface to display contents accordingly. Likewise, it could be in principle possible to speed the learning processes with unfamiliar interfaces by showing specific types of interface-hints depending on the user's history of interactions. In the case of non-experts, one could provide contextual cues to facilitate the development or discovery of strategies that have proven successful for experts. Alternatively, because the model is capable of detecting quite robustly when a user is not learning (i.e., when the random strategy dominates), the interface could choose to display challenging options or hints in order to “wake up” the user in such cases.

It is notorious that individual differences in behavior have rarely been the target for computational modeling or, in some cases, even treated as a nuisance (Karwowski and Cuevas, 2003). This is all the more surprising given the importance that recognizing individual differences can have for such disparate but fundamental domains as educational interventions (Detterman and Thompson, 1997; Phillips and Lowenstein, 2011; Melby-Lervag et al., 2012) or human performance studies (Van Dongen, 2006; Parasuraman and Jiang, 2012; Goel et al., 2013). We surmise that the framework presented here could be used to tackle this issue because it is built upon modular strategies whose combination can capture a diversity of behaviors, even among learners (see for instance Figure 5A vs. Figure 5B in which two different types of learners can be clearly distinguished). We believe that much more research is needed on the issue of modeling behavior in a way that takes into account the specifics of individuals, in addition to average cases or proof of concept approaches (Smith et al., 2014).

## Author contributions

CM participated in the experimental design, performed the experiment and acquired, analyzed the data and contributed with the interpretation of the results and the writing of the manuscript. DC designed the experiment, contributed with experimental and data recording equipment, contributed to the interpretation of the results and wrote and edited the manuscript. RV contributed with the analysis of the data and with the interpretation of the results as well as revising critically the manuscript. VL contributed to the experimental design and interpretation of the results, participating in the writing and revision of the manuscript. DM contributed with the interpretation of the data, the formalization of the model, and the critical revision of the manuscript.

### Conflict of interest statement

The authors declare that the research was conducted in the absence of any commercial or financial relationships that could be construed as a potential conflict of interest.
